# Deep characterization of human γδ T cell subsets defines shared and lineage-specific traits

**DOI:** 10.3389/fimmu.2023.1148988

**Published:** 2023-03-31

**Authors:** Marta Sanz, Brendan T. Mann, Paul L. Ryan, Alberto Bosque, Daniel J. Pennington, Holger Hackstein, Natalia Soriano-Sarabia

**Affiliations:** ^1^ Department of Microbiology, Immunology and Tropical Medicine, George Washington University, Washington, DC, United States; ^2^ Centre for Oral Immunobiology and Regenerative Medicine, Institute of Dentistry, Barts and The London School of Medicine and Dentistry, Queen Mary University of London, London, United Kingdom; ^3^ Centre for Immunology, Blizzard Institute, Barts and The London School of Medicine and Dentistry, Queen Mary University of London, London, United Kingdom; ^4^ Department of Transfusion Medicine and Hemostaseology, Friedrich-Alexander University Erlangen-Nuremberg, University Hospital Erlangen, Erlangen, Germany

**Keywords:** innate immunity, TCR - T cell receptor, gamma delta (γδ) T cells, Vdelta1, Vdelta2

## Abstract

Under non-pathological conditions, human γδ T cells represent a small fraction of CD3^+^ T cells in peripheral blood (1-10%). They constitute a unique subset of T lymphocytes that recognize stress ligands or non-peptide antigens through MHC-independent presentation. Major human γδ T cell subsets, Vδ1 and Vδ2, expand in response to microbial infection or malignancy, but possess distinct tissue localization, antigen recognition, and effector responses. We hypothesized that differences at the gene, phenotypic, and functional level would provide evidence that γδ T cell subpopulations belong to distinct lineages. Comparisons between each subset and the identification of the molecular determinants that underpin their differences has been hampered by experimental challenges in obtaining sufficient numbers of purified cells. By utilizing a stringent FACS-based isolation method, we compared highly purified human Vδ1 and Vδ2 cells in terms of phenotype, gene expression profile, and functional responses. We found distinct genetic and phenotypic signatures that define functional differences in γδ T cell populations. Differences in TCR components, repertoire, and responses to calcium-dependent pathways suggest that Vδ1 and Vδ2 T cells are different lineages. These findings will facilitate further investigation into the ligand specificity and unique role of Vδ1 and Vδ2 cells in early immune responses.

## 1 Introduction

Gamma delta (γδ) T cells comprise a lymphocyte population expressing an antigen-specific T cell receptor (TCR) arranged through somatic recombination within the thymus similar to αβ T cells (1). Human peripheral blood γδ T cell subpopulations are classified as Vδ1 and Vδ2 T cells based on the δ-chain usage (2, 3). Vδ2 T cells represent the majority of circulating γδ T cells whereas the Vδ1 subpopulation is rare in the blood of healthy adults (0.1-0.3% of T cells) and instead primarily reside in barrier tissues such as the gut mucosa, lungs, and female reproductive system (4, 5). Despite their recognized role in different immune settings, characterization of their specific development and antigen recognition functions remains the focal point of continuous investigation. Both subsets are capable of recognizing a variety of non-protein antigens although TCR-activating ligands for Vδ1 T cells remain poorly characterized with only a limited number identified for specific subsets or clones (6–8). In contrast, Vδ2 T cells recognize low-molecular-weight phosphorylated compounds referred to as phosphoantigens (P-Ags) (9–11) in an MHC-unrestricted manner involving interaction with Butyrophilins (12–14).

γδ T cells have been broadly described as innate-like, but recent studies have revealed phenotypic and functional heterogeneity both between and within Vδ1 and Vδ2 responses including subpopulations displaying characteristics aligned with adaptive immunity (15–19). Either Vδ1 or Vδ2 T cells transiently expand in response to infection or malignancy. Although they exhibit different functionality depending on the immunological context, characterizing the intrinsic features that distinguish subset-specific responses remains challenging (20). *Ex vivo* gene expression comparison of purified human Vδ1 and Vδ2 T cells has been limited due to the difficulty in isolating sufficient numbers for further characterization. Gene expression profiles of purified, *in vitro* expanded Vδ1 and Vδ2 T cell subsets revealed specific changes in approximately 50% of genes suggesting non-redundant roles following activation (21). Recent advances in single cell sequencing technologies have facilitated analyses on purified γδ T cell populations *ex vivo*, including a report from a CMV+ individual showing unique transcriptome signatures for Vδ1 cells and Vδ2 T cells (22). TCR repertoire analyses of each subset has also highlighted key differences including factors that drive their clonal expansion such as CMV responsive Vδ1+/Vδ2- clonotypes or microbial exposure during early life shaping the Vδ2 T cell compartment (23–25). Subset specific *in vivo* expansion can occur within different pathologies such viral (26, 27) or bacterial infection (28–30), cancer (31, 32), and inflammatory diseases (33). We hypothesized that Vδ1 and Vδ2 T cells bear unique similarities and differences that define their non-redundant roles in response to a given immunological challenge and distinguish them as separate lineages. Unravelling the specific role of each γδ T cell population is key to identifying their specific ligands and the pathways that regulate their responses. Deeper characterization may facilitate targeted modulation of their immune responses against infections and malignancy.

## 2 Materials and methods

### 2.1 Samples

Buffy coats were obtained from randomly selected healthy, volunteer donors after giving informed consent (approved by Ethics Committee, University of Giessen; file 05/00) from the Institute for Clinical Immunology and Transfusion Medicine (Justus-Liebig University, Giessen, Germany). Additional deidentified samples were obtained from the New York Blood Center (Long Island City, NY, USA) or the Gulf Coast Regional Blood Center (Texas). Daudi human Burkitt’s lymphoma cell line was obtained from the American type culture collection (ATCC).

### 2.2 Phenotypic and functional markers in γδ T cell populations

Multiparametric flow cytometry was performed using isolated PBMCs from 11 donors. Combinations of monoclonal antibodies (mAbs) were analyzed in three different panels for phenotyping γδ T cell populations (**Supplementary Table 1**). 5 x 10^5^ PBMCs were washed and stained with a viability dye (Zombie Aqua Fixable viability kit, BioLegend) for 15 minutes at room temperature. Cells were then washed and resuspended in staining buffer (PBS + 2% FBS) and surface stained with a core panel of mAbs against: CD3 (clone SK7), CD4 (clone SK3), CD8 (clone SK1), Vδ1 (clone REA173, Miltenyi Biotec), and Vδ2 (clone B6) to define T cell lineages in addition to either a panel of mAbs to characterize cytotoxicity and exhaustion: CD56 (clone 5.1H11), CD16 (clone 3G8), NKp30 (clone p30-15), NKp44 (clone P44-8), NKG2D (clone 1D11), PD-1 (clone EH12.2H7), TIGIT (clone A15153G), a panel defining tissue homing: CD103 (clone Ber-ACT8), α_4_β_7_ (clone Hu117 R&D Systems), CD161 (clone DX12 BD Biosciences), CCR6 (clone G034E3), CD69 (clone FN50), CXCR5 (clone J252D4), or a panel defining memory and activation: CD45RA (clone HI100), CD27 (clone M-T271), CCR7 (clone G043H7), CCR5 (clone J418F1), and HLA-DR (clone H243). Cells were incubated at for 20 minutes on ice in the dark, washed and fixed with 2% paraformaldehyde prior to acquisition on a BD LSRII Fortessa TM X-20 instrument (BD Biosciences) and analyzed using FlowJo v.10.8.1 (Tree Star). Unstained and fluorescence minus one (FMO) controls were run in parallel. T-distributed stochastic neighbor embedding (t-SNE) was conducted using concatenated CD3+Vδ1+ and CD3+Vδ2+ events from five separate donors acquired under the same compensated parameters.

### 2.3 Cytotoxicity assays

γδ T cell populations were sorted using a FACS-Aria II (BD) and coculture with the cancer cell line Daudi cells at a 1:1 effector:target ratio, and compared to a Daudi cells alone control, and γδ T cells alone control as previously described (34). For each staining, cells were incubated in 100% FBS for 10minutes to avoid unspecific binding of the antibodies and collected in enriched media containing 20% FBS. Briefly, for degranulation assays cells were incubated with CD107a in the presence of Brefeldin A (Biolegend) for 4 hours, washed, permabilized, and stained with mAbs against granzyme B (GzmB) and perforin for 30 min. To analyze Daudi cell killing, the cells were incubated for 15 min with viability dye 7-AAD (BD Biosciences) and analyzed using FlowJo v10.8.1. Daudi cell killing mediated by Vδ1 or Vδ2 T cells is presented as normalized to the Daudi cells cultured alone condition.

### 2.4 Immune TCR repertoire sequencing assays

Immune TCR Repertoire Sequencing Assay was performed as we previously described (34). Briefly, one million PBMC were pelleted and stored at −80°C. Total RNA was extracted (Qiagen) and genomic libraries were made following Archer VariantPlex Protocol for Illumina (Archer, Cat: SK0096) in conjunction with the corresponding target enrichment panel utilizing molecular barcode adapters (MBCs). Samples were then multiplexed together to increase sample diversity before running on a NextSeq 500 and were analyzed using Archer Analysis software.

### 2.5 FACS-sorting of γδ T cell populations

Due to the low frequency of circulating γδ T cells (1-10% of total CD3+ T cells), four buffy coats from healthy volunteers were screened for Vδ1 and Vδ2 T cells to ensure adequate numbers for microarray analysis. PBMCs from the selected donors were isolated by Ficoll-centrifugation. Utilizing an optimized a FACS-sort method (35) developed by our group enabled isolation of highly purified γδ T cell subsets (FACS –Aria II, BD) to perform gene expression analysis. Briefly, γδ T cell populations were selected as CD3+ Vδ1+ or Vδ2+ lacking the expression of αβTCR, CD14, CD19, BDCA-1, BDCA-2, BDCA-4. Cells were washed two times and resuspended in an optimized PBS sort buffer containing 5mM EDTA and 25mM Hepes. Cells were sorted in a FACS-Aria II instrument (BD) and collected in complete RPMI with 15% pooled human AB serum. Purity of the isolated populations was greater than 99% (**Supplemental Figure 1**). Cells were then centrifuged at 1600rpm for 15min, resuspended in RNAlater and stored at -80°C until RNA from all samples was extracted in parallel.

### 2.6 Whole genome microarray analysis

#### 2.6.1 RNA isolation, quality control and cDNA synthesis

Samples were shipped to Miltenyi Biotec GmbH for RNA isolation and microarray analysis. RNA was isolated using Trizol, and quality control on total isolated RNA was performed using the Agilent 2100 Bioanalyzer expert software that allows for visual control and generation of an RNA integrity Number (RIN) for integrity and overall quality of the samples (36). SuperAmp RNA amplification was performed according to Miltenyi Biotec’s procedure. Briefly, the amplification is based on a global PCR protocol using mRNA-derived cDNA. mRNA was isolated *via* magnetic bead technology. Amplified cDNA samples were quantified using the ND-1000 Spectrophotometer (NanoDrop) showing a consistent 260/280 ratio of 1.8 across all samples. The integrity of the cDNA was checked *via* the Agilent 2100 Bioanalyzer platform (Agilent Technologies). The results of the Bioanalyzer run are visualized in a gel image and an electropherogram using the Agilent 2100 Bioanalyzer expert software. The average length of the highly amplified cDNA products ranged between 200–1,000 bp.

#### 2.6.2 Agilent whole human genome oligo microarray

250 ng of each of the cDNAs were used as template for Cy3 labeling which was performed according to Miltenyi Biotec’s undisclosed protocol. The Cy3- labeled cDNAs were hybridized overnight (17 hours, 65°C) to an Agilent Whole Human Genome Oligo Microarrays 4 x 44K using Agilent’s recommended hybridization chamber and oven. Finally, the microarrays were washed once with 6x SSPE buffer containing 0.005% N-lauroylsarcosine for 1* min* at room temperature followed by a second wash with pre-heated 0.06x SSPE buffer (37°C) containing 0.005% N-lauroylsarcosine for 1* min*. Fluorescence signals of the hybridized Agilent Microarrays were detected using Agilent’s Microarray Scanner System (Agilent Technologies). The Agilent Feature Extraction Software (FES) was used to read out and process the microarray image files. For determination of differential gene expression FES derived output data files were further analyzed using the Rosetta Resolverâ gene expression data analysis system (Rosetta Biosoftware). Ratios were calculated by dividing sample signal intensity through control signal intensity (automated data output of the Resolverâ system).

### 2.7 Intracellular expression of NFAT and AhR

PBMCs from seven donors were enriched for γδ T cells by depleting the αβTCR+ cells (Stem Cell Technologies). 5x10^5^ untouched cells were incubated in the absence or presence of 800nM of the calcineurin inhibitor Cyclosporin *A* (CsA) or 10 µM of AhR inhibitor CH-223191 (both from Sigma-Aldrich) for 30 minutes at 37°C. Cells were then incubated for three and 24 hours in the presence of 1µg/mL Ionomycin (Stem Cell) or 10 µg/mL of a mAb targerting CD3δ (clone 5A6.E9, Millipore Sigma). After incubation, cells were washed and stained with a viability dye (Zombie Aqua Fixable viability kit, BioLegend) for 15 minutes at room temperature. Surface staining was performed as above using CD3 (clone SK7), Vδ2 (clone B6), CD69 (clone FN50) (all from Biolegend) and Vδ1 (clone REA173, Miltenyi Biotec). Cells were fixed and permeabilized using the FoxP3 Fixation/Permeabilization kit (Invitrogen), and stained with 100µL permeabilization buffer containing mAbs against AhR (Clone FF3399, Invitrogen) and NFATc1 (clone 7A6, Biolegend) and incubated at 4°C overnight. Cells were washed with 2mL of permeabilization buffer, acquired on a BD LSRII Fortessa TM X-20 instrument (BD Biosciences) and analyzed using Flowjo (FlowJo v.10.8.1).

### 2.8 Statistical analysis

Non-parametric two-sided tests were used for the study. Comparisons between groups were performed by Mann-Whitney U tests while repeated measures were compared using a Wilcoxon matched-pairs signed rank test. Correlation analysis were performed using Spearman’s tests. Analysis were performed using the *IBM* SPSS Statistics 26 software or Prism-GraphPad v.9. The microarray data analysis was provided by Miltenyi Biotec.

## 3 Results

### 3.1 Frequency of Vδ1 and Vδ2 T cells

**Figure 1:**
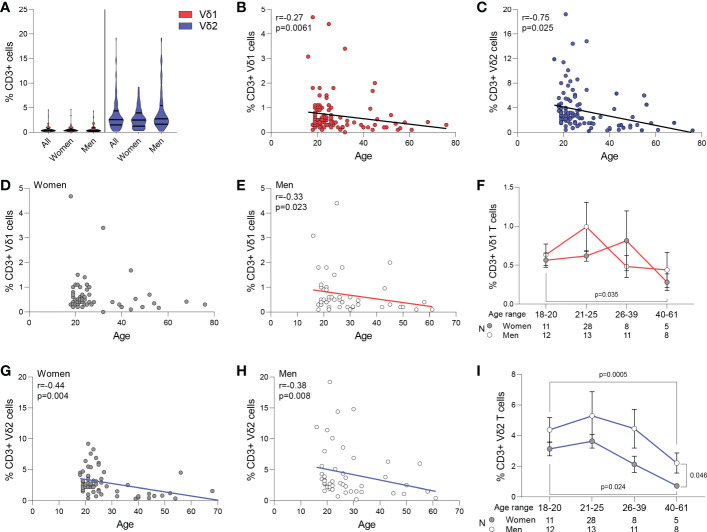
Frequency of Vδ1 and Vδ2 T cells. Circulating frequency of γδ T cell populations in 108 healthy blood donor volunteers. **(A)** Violin plots show frequency of Vδ1 and Vδ2 T cells in the entire study cohort, with no differences between women and men. A median of 0.4% (range 0-4.4) were Vδ1 T cells, and a median of 2.6% (range 0.3-19.20) were Vδ2 T cells (p<0.001). Correlation of age with frequency of **(B)** Vδ1 and **(C)** Vδ2 T cells. **(D)** Vδ1 T cells frequency was maintained with aging in women and **(E)** decreased in men. **(F)** Frequencies of Vδ1 T cells between women and men in different ranges of age. Mean ± SEM is shown. Frequency of Vδ2 T cells decreased with age in both **(G)** women and **(H)** men. **(I)** Vδ2 T cell numbers decreased in both women and men, with a more pronounced decay in women after 40 years of age compared to men Mean ± SEM is shown. Vδ1 T cells are represented in red, Vδ2 T cells are represented in blue. Women are represented as filled circles and men as open circles. Correlation analysis by Spearman’s test.

Circulating frequency of γδ T cell populations were quantified in 108 healthy blood donor volunteers recruited at the Institute of Clinical Transfusion Medicine, Germany and The George Washington University. Donors had a median age of 23 (range 18-61) years old, and 52 (54%) donors self-reported to be women while 44 (46%) self-reported to be men. The race/ethnicity of the donors was not recorded at the time of blood donation for the donors recruited in Germany. Among total CD3+ T cells, a median of 0.4% (range 0-4.4) were Vδ1 T cells, and a median of 2.6% (range 0.3-19.20) were Vδ2 T cells (p<0.001, **Figure 1A**), and had a similar representation between women and men (p>0.05, **Figure 1A**). Frequency of both Vδ1 and Vδ2 T cells inversely correlated with age (p=0.034 and p=0.001, **Figures 1B, C**, respectively). Further analysis revealed that the frequency of Vδ1 T cells was maintained with aging in women (**Figure 1D**), and only decreased in men and (**Figure 1E**). However, the frequency of Vδ2 T cells decreased with age in both women (**Figure 1G**) and men (**Figure 1H**). A comparison of cell frequencies in different ranges of age showed comparable frequencies of Vδ1 T cells between women and men, and a decrease from the twenties to the forties in women (**Figure 1F**). Finally, Vδ2 T cell numbers decreased in both women and men, with a more pronounced decay in women after 40 years of age compared to men (**Figure 1I**).

### 3.2 Phenotype of Vδ1 and Vδ2 T cells

**Figure 2:**
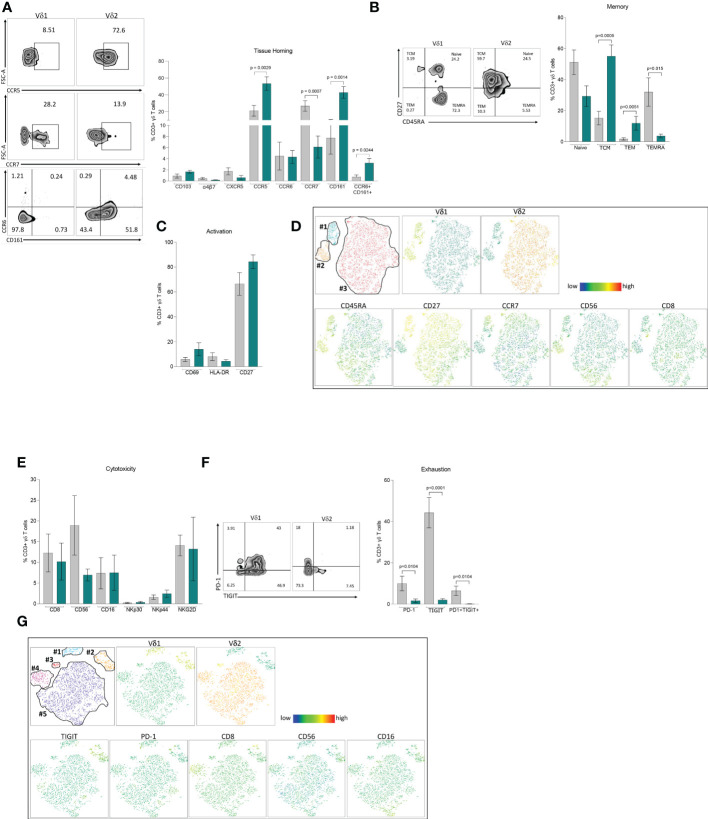
Phenotype of Vδ1 and Vδ2 T cells. Multiparametric flow cytometry in PBMCs from 11 healthy donor volunteers. **(A)** Tissue-homing receptors. **(B)** Memory populations defined as Naïve (CD45RA+CD27+), TCM, central memory (CD45RA-CD27+), TEM, effector memory (CD45RA-CD27-) and TEMRA, (CD45RA+CD45+CD27-) **(C)** Activation markers, CD69, HLA-DR and CD27. **(D)** t-SNE analysis of memory markers based on Vδ1 and Vδ2 T cells. **(E)** Cytotoxicity markers CD8, CD56, CD16, NKp30, NKp44 and NKG2D. **(F)** Expression of immune exhaustion markers, PD-1, TIGIT and co-expression of PD1/TIGIT. **(G)** t-SNE analysis of cytotoxic markers based on Vδ1 and Vδ2 T cells expressing TIGIT. Mean ± SEM is shown; Mann-Whitney U test p-values < 0.05 are presented. Vδ1 T cells are shown in grey and Vδ2 T cells are shown in green.

To understand phenotypic differences between each γδ T cell subset, we performed multiparametric flow cytometry using three comprehensive panels for markers of tissue homing, memory, activation, exhaustion, and cytotoxicity. Based on their distinct anatomical distribution, we hypothesized that peripheral γδ T cells differentially express tissue homing receptors. Although Vδ1 T cells appear in higher frequencies in the gastrointestinal tract, there were no differences in the frequency of either subset expressing markers associated with migration (CCR6 and α_4_β_7_) or residency (CD103) within the intestinal mucosa (**Figure 2A**). Instead, the most pronounced differences were observed in chemokine receptors involved in tissue homing CCR7 and CCR5 with higher expression in Vδ1 and Vδ2 respectively. Additionally, we found a higher frequency of Vδ2 T cells coexpressing CCR6 and CD161 which has been associated with a T_H_17 phenotype under pathological conditions (37–39). This data is suggestive of potential differences in the effector/memory phenotypes of each subset. In agreement with previous studies, the majority of Vδ1 T cells express CD45RA and can be categorized as primarily Naïve (CD45RA+CD27+) or TEMRA (CD45RA+CD27-) when defined by the coexpression of the costimulatory marker CD27 (**Figure 2B**) (15, 40). This is juxtaposed against Vδ2 T cells that have a predominate effector phenotype with higher frequencies classified as TCM (CD45RA-CD27+) and TEM (CD45RA-CD27-). Despite these pronounced differences in memory, there were no differences in early or late activation markers as well as single expression of CD27 between the two subsets (**Figure 2C**).

To assess the relative expression of each marker and potential heterogeneity within effector/memory γδ T cell subsets, we performed t-distributed stochastic neighbor embedding (t-SNE) analysis (**Figure 2D**). Vδ1 T cells formed two distinct clusters featuring either a Naïve (cluster #1) or TEMRA (cluster #2) phenotype, coincident with the individual expression of CD45RA and CD27, and similar to our previous analysis (**Figure 2B**). By contrast, Vδ2 T cells appeared as a single cluster (cluster #3) without a clear demarcation based on memory phenotype. Cluster #1 not only had the highest relative expression of CD27, but also contained cells expressing CCR7. Notably, this segment of cluster #1 had lower expression of cytotoxic markers CD8 and CD56 indicative of a less cytotoxic phenotype. While both markers were evenly distributed in Vδ2 T cells, CD56 expression in particular was more prominent in the TEMRA-like cluster #2 of Vδ1 T cells (**Figure 2D**).

Phenotyping analysis of different cytotoxic markers showed comparable single expression of CD8, CD56, CD16, NKp30, NKp44 and NKG2D between Vδ1 and Vδ2 T cells (**Figure 2E**). Analysis of immune exhaustion markers revealed higher frequencies of single and double expression of Programmed cell death protein 1 (PD-1) and T cell immunoreceptor with Ig and ITIM domains (TIGIT) in Vδ1 T cells compared to Vδ2 T cells (**Figure 2F**). Next, we assessed whether PD-1 or TIGIT expression identified discrete cytotoxic γδ T cell subpopulations. t-SNE analysis identified two distinct Vδ1 T cell clusters (#1 and #2), and three clusters for Vδ2 T cells (#3, #4 and #5, **Figure 2G**). TIGIT, but not PD-1, represented a distinguishing marker for Vδ1 T cells from cluster #2 (**Figure 2G**). This cluster had notably higher relative expression of CD8, CD56, and CD16 suggesting that Vδ1 T cells expressing TIGIT identify a discrete population displaying a more cytotoxic profile compared to low expressing or TIGIT negative Vδ1 T cells. By comparison, expression of cytotoxic markers within Vδ2 T cell clusters did not coincide with higher PD-1 or TIGIT expression. Instead, each Vδ2 T cell cluster had unique cytotoxic expression profiles. Cluster #3 not only had minimal expression of each cytotoxic marker but the Vδ2 TCR was expressed at a lower frequency. Cluster #4 had markedly higher CD8 expression. Lastly, cluster #5 had intermediate expression of CD8 and CD56, with a portion of cells displaying high CD16. These results show a diverse composition of phenotypes within γδ T cell populations that coincide with a discrete distribution of cytotoxicity and effector markers. Whether the specific immune functions associated with these phenotypes are regulated through distinct mechanisms in each subset warrants further investigation.

### 3.3 Cytolytic function of Vδ1 and Vδ2 T cells

**Figure 3:**
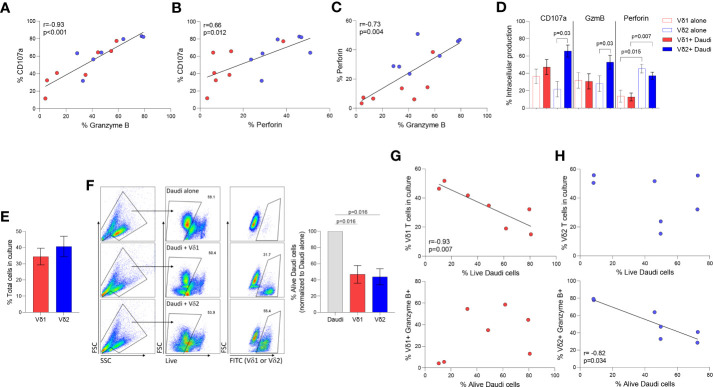
Cytotoxic function of γδ T cell subsets. Daudi Burkitt’s lymphoma cell line was used to compare Vδ1 and Vδ2 T cells cytolytic function in seven healthy donor volunteers. Correlation between different intracellular markers upon coculture of Vδ1 (red) and Vδ2 (blue) T cells and Daudi cells, **(A)** CD107 and GzmB, **(B)** CD107a and perforin, and **(C)** Perforin and GzmB. **(D)** Expression of CD107a, GzmB and perforin in Vδ1 and Vδ2 T cells. **(E)** Comparison of the total number of Vδ1 and Vδ2 T cells during co-culture with Daudi cells (). **(F)** Comparison of Daudi cell killing capacity of Vδ1 or Vδ2 T cells. Correlation of killing capacity with **(G)** Vδ1 T cells’ frequency in culture, and GzmB production. Correlation of killing capacity with **(H)** Vδ2 T cells’ frequency in culture and GzmB production (r_s_=-0.82, p=0.034). Mean ± SEM is shown. Daudi cell killing analyzed by Wilcoxon matched-pairs rank test. Correlations by Spearmans’ test.

Degranulation and cytotoxicity assays were performed as we previously described (34). Daudi Burkitt’s lymphoma cell line was used to compare Vδ1 and Vδ2 T cells cytolytic function in seven healthy donor volunteers. Intracellular Granzyme B (GzmB) and perforin were quantified upon coculture with Daudi cells in degranulation assays using CD107a. The three markers of cytotoxic function positively correlated between each other (**Figure 3A**, CD107a v. GzmB p <0.001, **Figure 3B**, CD107a v. Perforin p= 0.01, and **Figure 3C**, Perforin v. GzmB p= 0.004). As previously reported, Daudi cells did not induce the activation of Vδ1 T cells (refs), as confirmed by the lack of induction of either of these markers upon coculture (**Figure 3D**). On the contrary, CD107a and GzmB, but not perforin, were induced upon coculture in Vδ2 T cells. CD107a and GzmB production were comparable between Vδ1 and Vδ2 T cells (p=0.26 and p=0.16, respectively, **Figure 3D**) and perforin production was higher in Vδ2 T cells compared to Vδ1 T cells (p=0.007, **Figure 3D**), despite similar number of effector cells present in the culture (p=0.46, **Figure 3E**).

The frequency of alive Daudi cells upon coculture with γδ T cell populations was similar (mean of alive Daudi cells of 46.87% p= 0.016 *vs*. 43.83% p= 0.016, respectively **Figure 3F**). The frequency of alive Daudi cells, was associated with the number of Vδ1 T cells harvested after the coculture (r_s_=-0.93, p=0.007, **Figure 3G**). Conventional cytotoxic granule production in Vδ1 T cells was not induced by Daudi cells nor correlated with the frequency of perforin or GzmB positive cells (p=0.840 and p=0.302 respectively, **Figure 3G**). This suggests the possibility of Vδ1 T cells influencing viability by targeting alternative pathways (24). On the contrary, the frequency of alive Daudi cells was associated with a higher production of GzmB (r_s_=-0.82, p=0.034, **Figure 3H**), but not with the number of Vδ2 T cells present in the culture (p=0.713).

### 3.4 Gene expression analysis

**Figure 4:**
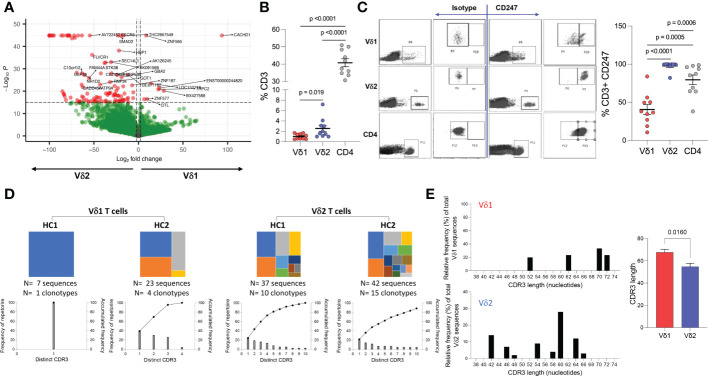
Whole genome and TCR sequencing. *Ex vivo* whole genome microarray data analysis of highly purified resting Vδ1 and Vδ2 T cells from four healthy donor volunteers. **(A)** Volcano plot displaying differentially expressed genes in Vδ1 and Vδ2 T cells (p<0.01 cut-off and 2-fold differential signal intensities). **(B)** Frequencies of Vδ1, Vδ2, and CD4 T cell amongst total CD3+ T cells in 10 donors used to corroborate **(C)** the differences in CD247 gene expression differences at the protein level by flow cytometry. **(D)** TCR repertoire sequencing in two healthy donor volunteers showing unique clonotypes as a proportion of the total repertoire (top panels). Lower graphs represent TCR occupancy of the top ten most frequent clonotypes. **(E)** Relative differences in CDR3 length usage amongst Vδ1 and Vδ2 clonotypes.

To identify differences in transcriptional profile between Vδ1 and Vδ2 T cells, whole genome microarray data analysis was performed using *ex vivo* highly purified resting Vδ1 and Vδ2 T cells from four healthy donors (**Supplemental Figure 1**). 1,749 genes were upregulated in Vδ1 T cells while 3,367 were upregulated in Vδ2 T cells, at the p<0.01 cut-off and 2-fold differential signal intensities (**Supplemental Table 1** and **Supplemental Figure 2**). The expression of the Cache domain containing 1 (*CACHD1*) gene was upregulated by 93.4-fold in Vδ1 T cells compared to Vδ2 T cells. The vascular endothelial growth factor B (*VEGFB*) and the *EXOC7* genes were 100-fold upregulated in Vδ2 T cells compared to Vδ1 T cells, suggesting specific expression of these genes in each population (**Figure 4A** and **Supplemental Table 1**).

Both γδ T cell subsets differentiate in the thymus and share several features including the expression of a TCR-CD3 complex. Upon TCR engagement, specific motifs in the CD3 chains CD3δ, CD3ε, CD3γ and CD3ζ (CD247) become phosphorylated by Scr family protein tyrosine kinases LCK and FYN. Phosphorylation of CD247 further induces phosphorylation of ζ-chain of T cell receptor associated protein kinase 70 (ZAP70), and activate downstream signaling pathways (41). Here, we found similar gene expression of CD3 chains δ-, ε-, and γ-, LCK, ZAP70, and LAT (**Table 1** and **Supplemental Table 1**) in Vδ1 and Vδ2 T cells. However, CD247 gene expression was upregulated by 57.9-fold in Vδ2 T cells compared to Vδ1 T cells, and protein expression was further analyzed by intracellular flow cytometry in 10 healthy donors. Vδ1, Vδ2 and CD4 T cell frequencies within total CD3+ T cells in this subgroup of donors were the expected (median frequency of 0.9%, 1.8% and 39.7%, respectively, **Figure 4B**). CD247 expression was the highest within Vδ2 T cells followed by CD4+ T cells and finally Vδ1 T cells (median of 99.75%, 86.4%, and 37.25% respectively, **Figure 4C**). This result provides evidence of a critical differential feature in the TCR-CD3 complex between γδ T cell subsets possibly associated with a readily effector function in Vδ2 T cells.

**Table 1:** Relative expression of the TCR components..

Gene	Fold-change Relative Expression*	p-value	NCBI Accession #
CD3γ	-2.281	0.167	NM_000073
CD3δ	-2.116	0.371	NM_000732
CD3ϵ	-1.435	0.572	NM_000733
**CD3**ζ	**-57.999**	**1.02E-16**	**NM_198053**
LCK	-1.288	0.669	NM_005356
ZAP70	1.065	0.915	NM_001079
LAT	-1.678	0.287	NM_032463
LAT2	1.201	0.724	NM_198053

*Negative fold-change values represent genes with higher relative expression in Vδ2 T cells.

Finally, analysis of the TCR repertoire in two healthy donor volunteers showed that Vδ1 T cells may have reduced repertoire diversity compared to Vδ2 T cells, evidenced by a lower TCR occupancy in the latter (**Figure 4D**). Analysis of the complementary-determining region (CDR)3 repertoire revealed shorter sequences in Vδ2 T cells compared to Vδ1 T cells (**Figure 4E**), indicative of subset-specific ligand recognition.

### 3.5 Transcription factors associated with upregulated genes within Vδ1 and Vδ2 T cell subsets

**Figure 5:**
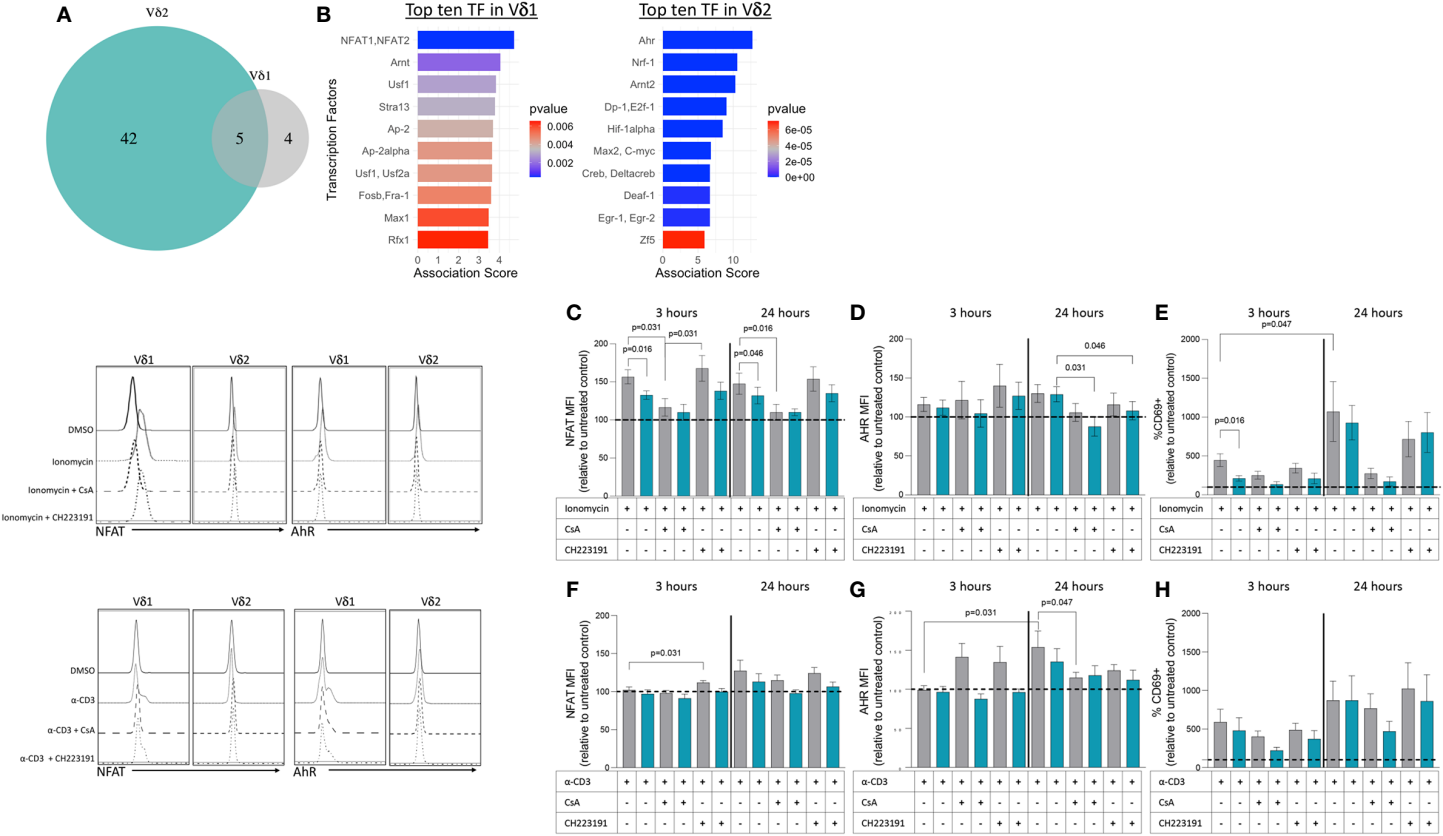
Transcription factors associated with upregulated genes within Vδ1 and Vδ2 T cell subsets. **(A)** The Predicting associated transcription factors from annotated affinities (PASTAA) software revealed a list of four transcription factors (TFs) associated with Vδ1 T cells, five TFs common to both subpopulations, and 42 associated with Vδ2 T cells. **(B)** Top TFs associated with Vδ1 and Vδ2 T cells, are shown. Upon stimulation with ionomycin **(C)** NFAT **(D)** AhR and **(E)** CD69 or α-CD3 monoclonal antibody **(F)** NFAT **(G)** AhR and **(H)** CD69 expression were analyzed in Vδ1 and Vδ2 T cells after 3 and 24 hours of exposure in the presence or absence of inhibitors of NFAT (cyclosporine A, CsA) or AhR (CH223191). Wilcoxon Rank-Sum test were used to compare the data. Normalized mean fluorescence intensity (MFI) and frequency of CD69 expression to the untreated or DMSO controls are presented.

We next wanted to address whether the differences in transcriptional profiles between Vδ1 and Vδ2 T cells were associated with the activity of specific transcription factors (TF). To that end, we used the software Predicting Associated Transcription factors from Annotated Affinities (PASTAA) (42). PASTAA predicts the TF associated with genes upregulated or downregulated in a sample. PASTAA revealed a list of four transcription factors (TFs) exclusively associated with Vδ1 T cells, five TFs common to both subpopulations, and 42 associated with Vδ2 T cells (**Figure 5A**). Interestingly, the TF NFAT1 and NFAT2 were highly associated with the transcriptional profiles of Vδ1 while Ahr was highly associated with Vδ2 (**Figure 5B**). On the other hand, the TF AhR nuclear translocator (ARNT) was shared by the two γδ T cell subsets, evident of common signaling component, in addition to TFs that act as global regulators of cell proliferation or apoptosis such as AP-2α, MYC-associated factor X (MAX), and upstream stimulatory factor 1 (USF1) (**Figure 5A**). To confirm these findings, modulation of the expression of NFAT and AhR was analyzed by intracellular flow cytometry after exposure to both TCR-dependent and TCR-independent stimuli in PBMCs from seven healthy donor volunteers. Cells were exposed to ionomycin that activates NFAT (43) or an activating dose of a CD3-specific antibody in the presence or absence of inhibitors of NFAT (cyclosporine A (CsA) or AhR (CH223191). Upon treatment with ionomycin, NFAT expression increased in both subsets compared to DMSO controls but was higher in Vδ1 T cells (**Figure 5C**). This effect persisted 24 hours following stimulation and was abrogated in the presence of CsA. Similarly, the expression of AhR increased in both subsets compared to media-only control, but there were no differences between Vδ1 and Vδ2 T cells. An effect from CH223191 at reducing AhR expression was only observed in Vδ2 T cells upon 24 hours of treatment with ionomycin (**Figure 5D**). NFAT expression in Vδ1 T cells was accompanied by a concomitant increase in the frequency of cells expressing the early activation marker CD69 (**Figure 5E**). While the frequency of CD69+ Vδ1 T cells was higher than Vδ2 T cells three hours after ionomycin treatment, the two populations had comparable expression 24 hours after stimulation. Conversely, stimulation through the CD3 complex did not induce overall changes in NFAT (**Figure 5F**) but did increase AhR expression in Vδ1 T cells 24 hours after treatment (**Figure 5G**). Although we observed a similar increase of AhR in Vδ2 T cells, this did not reach statistical significance. Both subsets had comparable increases in CD69 expression versus untreated controls indicating the differences in AhR were not due to disparities in activation (**Figure 5H**). These data support the findings from the PASTAA analysis and highlight the potentially different mechanisms that regulate γδ T cell subset immune responses. Vδ1 T cells possess higher sensitivity to ionomycin-mediated calcium release leading to a rapid increase of NFAT expression and cell activation. In contrast, Vδ2 T cells showed lower ionomycin sensitivity and delayed activation suggesting the need for additional co-stimulatory signals or additional time.

## 4 Discussion

Our study provides fundamental differences between Vδ1 and Vδ2 T cell subsets that distinguishes them as distinct lineages with unique roles in the immune system. This includes phenotypic and transcriptional differences that may have direct implications on how they respond to stimuli. Here, we show a similar representation of both Vδ1 and Vδ2 T cell populations in 108 healthy donor volunteers according to self-reported sex. As expected, we observed an age-related decline in Vδ2 T cell frequencies for both women and men although decreased Vδ1 T cell frequency with aging was observed only in men (44–46). A previous report in a Japanese cohort did not find Vδ1 T cell reductions for either sex (47). Despite an absence of race/ethnicity data for this current study, previous work by our group and others have demonstrated a clear association between race and γδ T cell biology which may contribute to the observed reduction in Vδ1 T cell frequencies within our aging male donors (34, 44, 45).

In our study, Vδ1 and Vδ2 T cells were phenotypically distinguished by their skewed expression patterns of memory markers CD45RA and CD27. In line with previous reports, Vδ1 T cells from healthy individuals primarily display a naïve (CD45RA+CD27+) phenotype with a portion of cells expressing conventional lymphoid homing receptors such as CCR7 (16, 40). Additionally, we found higher expression of the cytotoxic marker CD56 within TEMRA (CD45RA+CD27-) Vδ1 T cells. Previous work has shown that circulating Vδ1 clonotypes displaying this phenotype expand as a consequence of CMV infection and have higher production of cytotoxic granules in a comparable manner to TEMRA αβ CD8 T cells (16, 48). On the contrary, Vδ1 T cell phenotypes found within malignant tissue tend to skew towards a TEM (CD45RA-CD27-) phenotype in direct response to the tumor (49–51). While our analyses are limited by the absence of donor CMV status, deeper phenotypical characterization of effector Vδ1 T cells both within circulation and tissue may provide further understanding behind their differentiation in response to a given challenge. When compared to Vδ1 T cells, Vδ2 T cells in our study predominately showed a TCM (CD45RA-CD27+) phenotype with ubiquitously high expression of CCR5 and the C-type lectin-like membrane receptor CD161 suggestive that they are primed to rapidly migrate into inflammatory tissue. This is in agreement with previous studies showing that CCR5 expression is a distinguishing feature of Vδ2 T cells (52, 53). In or study, not only did Vδ2 T cells have higher single expression of CD161, but also co-expressed CCR6 at a higher frequency than Vδ1 T cells. Beyond their direct roles in facilitating leukocyte trafficking to sites of inflammation, expression of these receptors distinguishes fetal-derived Vδ2 T cells that undergo a degree of effector differentiation prior to microbial exposure and persist into adulthood (54, 55). Co-expression of CD161 and CCR6 is a hallmark of T_H_17 effectors although Vδ2 T cells with this phenotype maintain little to no IL-17A production outside of specific pathologies (56–58). Moreover, transcription factors that drive T_H_17 differentiation such as hypoxia-inducible factor 1 (HIF-1α) and early growth response gene-2 (Egr-2) were associated with significantly upregulated genes in our Vδ2 T cell microarray analysis (**Supplemental Table 1**) (59, 60). Murine γδ T cells differentiate into either IFN-γ (γδ^IFN^) or IL-17A (γδ (17)) producing effectors early in during thymic development with discrete metabolic programming that influences their responses within tumor microenvironments (61–63). This pronounced effector dichotomy has not been observed in human subsets. Collectively our data suggests that circulating Vδ2 T cells are predominately antigen-experienced and exist in a ready state to rapidly migrate to sites of inflammation upon immunological challenge. Despite evidence of memory-like characteristics from expanded Vδ1 and Vδ2 T cell clones following antigen exposure, the mechanisms that govern the formation of immunological memory within these populations remains to be elucidated (64).

γδ T cells role in immunosurveillance is mediated by the recognition of stress-induced ligands either directly through the TCR or cognate receptors typically found on Natural Killer cells (65). This includes constitutive expression of Natural Killer Group 2 members (66, 67) and inducible expression of natural cytotoxicity receptors NKp30 and NKp44 (68, 69). Unlike the majority of other T lymphocyte lineages, γδ T cells also express Neural cell adhesion molecule (CD56) and FcγRIIIA (CD16), the latter of which affords them the ability to recognize and kill IgG bound target cells *via* antibody dependent cytotoxicity (34, 70). While we found comparable expression of these individual cytotoxic markers, incorporating exhausted phenotypes into our t-SNE analysis identified two distinct Vδ1 T cell populations based on the expression of TIGIT, that was not observed within Vδ2 T cells. This Vδ1 TIGIT+ population coincided with elevated expression of the cytotoxic markers CD8, CD16, and CD56. TIGIT acts as a negative regulator of lymphocyte cytotoxicity by competing against its co-stimulatory counterpart DNAX Accessory Molecule-1 (DNAM-1) (71). Dysregulation of the TIGIT/DNAM-1 axis or co-expression with other exhaustion or immune checkpoint markers has been associated with reduced cytotoxic function of γδ T cells within the context of disease (72, 73). Furthermore, while Vδ1 T cells express higher levels of TIGIT compared to the Vδ2 T cells in healthy individuals, TIGIT expression is elevated in highly differentiated Vδ1 T cells in CMV-seropositive donors (74). Blockade of TIGIT signaling with the mAb EOS-448 restored pro-inflammatory cytokine production within this population, highlighting the inhibitory role of this receptor similar to αβ T cells. Therefore, immune checkpoint inhibitors represent a promising approach to reinvigorate highly cytotoxic γδ T cell populations for therapeutic purposes (75, 76). An alternative possibility is that TIGIT is a biomarker of a potent cytotoxic effector population similar to a recent study that showed NKG2A+ Vδ2 T cells possess enhanced anti-tumor capabilities (77). In our t-SNE analyses, Vδ2 T cells predominately formed a large, singular cluster indicative of relatively uniform phenotypes. Interestingly, a smaller cluster of cells with reduced Vδ2 TCR expression mapped closer to Vδ1 T cells. Although the majority of Vδ2 T cells express a semi-invariant Vγ9Vδ2 TCR that recognizes P-Ags, approximately 4-5% display differential Vγ-chain pairings and lower relative Vδ2 expression (17). This Vγ9- population shares similar adaptive properties with certain Vδ1 T cell subsets such as a diverse TCR repertoire, phenotypic heterogeneity, as well as differentiation and clonal expansion in response to CMV infection (78).

Both γδ T cell subpopulations are potent cytotoxic effectors that release perforin and granzymes following MHC-independent recognition of target cells (6, 79–81). To further understand the functional differences between Vδ1 and Vδ2 T cell cytotoxicity, we performed killing assays in cocultures with Daudi Burkitt’s lymphoma cells which lack stable expression of MHC class I molecules (82, 83). Our results showed that both Vδ1 and Vδ2 T cells possess a similar capacity to kill Daudi cells *ex vivo* without prior stimulation, albeit through potentially different mechanisms. Despite comparable target-to-effector cell ratios, only the killing capacity of Vδ2 T cells was associated with GzmB production. Both cell types expressed higher frequencies of granule markers following coculture, but Vδ2 T cells specifically had significantly higher perforin production and a notably higher rate of degranulation as shown by surface CD107a expression. Although early studies showed that peripheral Vδ1 and Vδ2 T cell clones had differing capacities to respond to Daudi cells *in vitro* which is likely attributed to the distinct TCR ligands that each subset recognizes, Vδ1 T cell clones were primarily non-responsive to Daudi cells with the exception of a subpopulation co-expressing Vγ9 (84, 85). Our results are in accordance with more recent studies which found comparable cytotoxicity between each subset following *ex vivo* expansion with an anti-γδ TCR antibody and IL-2, suggesting Vδ1 T cells may require activating stimuli prior to coculture (86). Daudi cells produce endogenous p-Ags, which are specifically recognized by Vδ2 T cells through their TCR and stimulate proliferative and cytolytic responses without the need for prior exposure (87). The absence of MHC I molecules creates an additional “missing self” scenario which facilitates Vδ2 T cell recognition (88). Given the comparable frequency of each effector within their respective cocultures, our data indicates that Vδ1 T cells either lyse Daudi cells through alternative mechanisms or possess highly cytolytic subpopulations that were not detected in previous studies that relied on cloning methodology. Additional investigation into the role of the Vδ1 TCR or alternative modes of recognition could prove fruitful for identifying tumor-specific clones for therapeutic approaches.

The unique contributions of each γδ T cell subset have been substantiated by their divergent transcriptional responses to both TCR-specific and independent stimuli *in vitro* (21, 89, 90). Our microarray analysis of *ex vivo* gene expression of purified Vδ1 and Vδ2 T cells identified subset-specific transcriptional programs that may influence their responses. Among genes upregulated in Vδ1 T cells, the highest fold-changes were observed for transcripts encoding potentiators of ion exchange (*CACHD1, SLC35B4, NKAIN3, S100A7*), hormone receptors (*IRS4, PGR*), and signal transduction (*PFTK1, RERGL*). Meanwhile Vδ2 T cells have a prevalence of highly expressed transcriptional and translational factors that directly control cellular activation, proliferation, or differentiation (*NFKB1, FOXP1, EFTUD2, EPC1*). These data suggest regulation of Vδ1 and Vδ2 T cell responses may be mediated by distinct factors or mechanisms irrespective of their TCR specificity. Differences between our results and a previous study may reflect the effects of *ex-vivo* expansion using a γδ-TCR antibody prior to gene expression analysis (21). Specifically, some of the findings related to exclusive TLR expression on one subset *vs*. the other were not confirmed in our study (**Supplemental Table 1**) using resting highly purified γδ T cell populations. Critical differences between Vδ1 and Vδ2 T cells were related to the expression of the CD3-ζ chain, being less expressed by Vδ1 T cells suggesting that their TCR-mediated contribution to immune responses may be shared with other innate-like pathways recognizing a broader set of ligands. Alternatively, CD3ζ- Vδ1 T cells may instead rely on substitutive signaling receptors as observed in CD3ζ-deficient mice that expressed FcϵRIγ and showed differential responses to TCR and antigenic stimulation (91). In addition, Vδ1 and Vδ2 T cells displayed differing patterns in TCR diversity that is likely reflective of the specific antigens they recognize and effector responses over the course of the human lifespan. Despite a limited sample size, our TCR repertoire analysis is congruent with existing studies that show adult peripheral Vδ1 T cells are dominated by one or a few clonotypes favoring longer CDR3 lengths whereas Vδ2 T cells are comprised of a higher frequency of clones possessing shorter CDR3 lengths (16, 17).

PASTAA analysis (42) revealed NFAT and AhR as the top TFs associated with differentially expressed genes in Vδ1 and Vδ2 T cells, respectively. Since NFAT has a critical role in T cell activation, we analyzed the expression of these TFs through exposure to TCR-dependent and TCR-independent stimuli. Confirming the PASTAA findings, treatment with ionomycin increased NFAT expression and induced higher activation of Vδ1 T cells compared to Vδ2 T cells. Calcium/calcineurin-regulated NFAT1 is a key regulator of T cell activation, differentiation and development and in αβT cells it is activated by engagement of the TCR (92). Our finding of higher CACHD1 and other ion channels in Vδ1 T cells could also potentially explain the heightened sensitivity to ionomycin treatment by increasing the level of intracellular calcium (93). AhR is a ligand-activated transcription factor that operates in a cell-type specific manner and modulates tissue homeostasis in αβ T cells (94). One key deficiency in our understanding of γδ T cell immunobiology is the lack of comparable subsets in animal models, precluding exact comparison with human γδ T cell populations. In addition, although most of the mechanisms of αβTCR signaling are thought to be similar for the γδTCR, both TCRs display different structure and components potentially providing distinct features (95, 96). Current knowledge on AhR expression and function comes from studies performed in mice, which do not possess the same γδ T cell populations as humans (97). Despite the lack of studies on AhR in human γδ T cells, murine studies have shown that AhR is expressed in all γδ T cell subsets and is critically required to maintain large populations of T lymphocytes at mucosal sites including intraepithelial lymphocytes of the intestine and skin (98, 99). AhR is essential for IL-22 production within murine γδ (17) T cells and cellular uptake of exogenous ligands is facilitated by CD69 (100, 101). We detected changes in AhR protein expression in Vδ1 but not Vδ2 T cells following TCR-CD3 complex stimulation, indicating a potential connection between antigen recognition and production of this transcription factor.

The dissection of lymphocyte subpopulations continues to reveal both the richness and robustness of the human immune system. Despite a growing appreciation for the contribution of γδ T cells in responding to infection and malignancy, there has been a lack of studies aimed at distinguishing individual subsets in humans. Our study outlines the key differences in peripheral Vδ1 and Vδ2 T cells *ex vivo*. We found differences in terms of their phenotypes, transcriptomes, and functional responses that indicate each subset belongs to a distinct cell lineage. The collective differences in these features likely reflect the unique stimuli and mechanisms that regulate peripheral human γδ T cell subsets. Current literature suggests Vδ1 T cell characteristics are primarily shaped by exposure to pathogens and disease encountered well after birth whereas Vδ2 T cells begin differentiating in the earliest stages of life (55, 102). Thymic programming also directly impacts γδ T cell effector metabolism and highlights the need for additional investigation into how this may influence each subset (63). Our findings will facilitate further investigation into the specific ligands each subset recognizes, the development of their effector responses, and their individual roles in the overall immune response.

## Data availability statement

The datasets presented in this study can be found in online repositories. The names of the repository/repositories and accession number(s) can be found here: GSE224362 (GEO).

## Author contributions

NS-S conceived and designed the study and wrote the manuscript. MS and BM performed research and wrote the manuscript. PR and DP performed TCR analysis and edited the manuscript. HH and AB analyzed the microarray data and edited the manuscript. All authors contributed to the article and approved the submitted version.
