# Supplementary material for: Deep characterization of human γδ T cell subsets defines shared and lineage-specific traits
Source: Front Immunol. 2023 Mar 31;14:1148988. doi: 10.3389/fimmu.2023.1148988 (PMC10102470; doi:10.3389/fimmu.2023.1148988)
Supplement: Supplementary file 2 [file DataSheet_2.docx]

Supplementary Material

**Deep Characterization of Human γδ T Cell Subsets Defines Shared and Lineage-Specific Traits**

Marta Sanz ^1†^, Brendan T. Mann^1†^, Paul L. Ryan ^2^, Alberto Bosque^1^, Daniel J. Pennington^3^, Holger Hackstein^4^, Natalia Soriano-Sarabia^1*^.

*** Correspondence:**Natalia Soriano-Sarabia, PhD, [nataliasorsar@gwu.edu](mailto:nataliasorsar@gwu.edu)


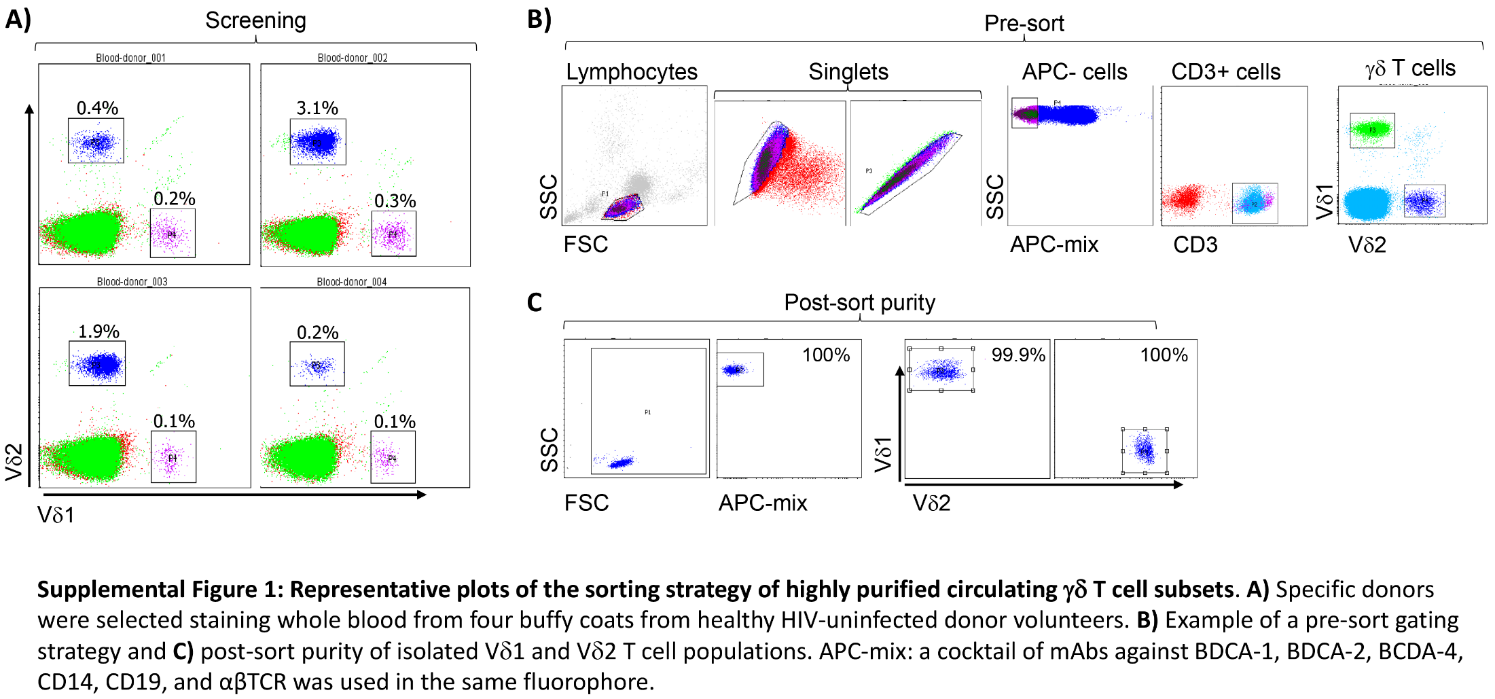


**Supplemental Figure 1.** Representative plots of the sorting strategy of highly purified circulating γδ T cell subsets. **A)** Specific donors were selected staining whole blood from four buffy coats from healthy HIV-uninfected donor volunteers. **B)** Example of a pre-sort gating strategy and **C)** post-sort purity of isolated Vδ1 and Vδ2 T cell populations. APC-mix: a cocktail of mAbs against BDCA-1, BDCA-2, BDCA-4, CD14, CD19 and αβ TCR was used in the same fluorophore


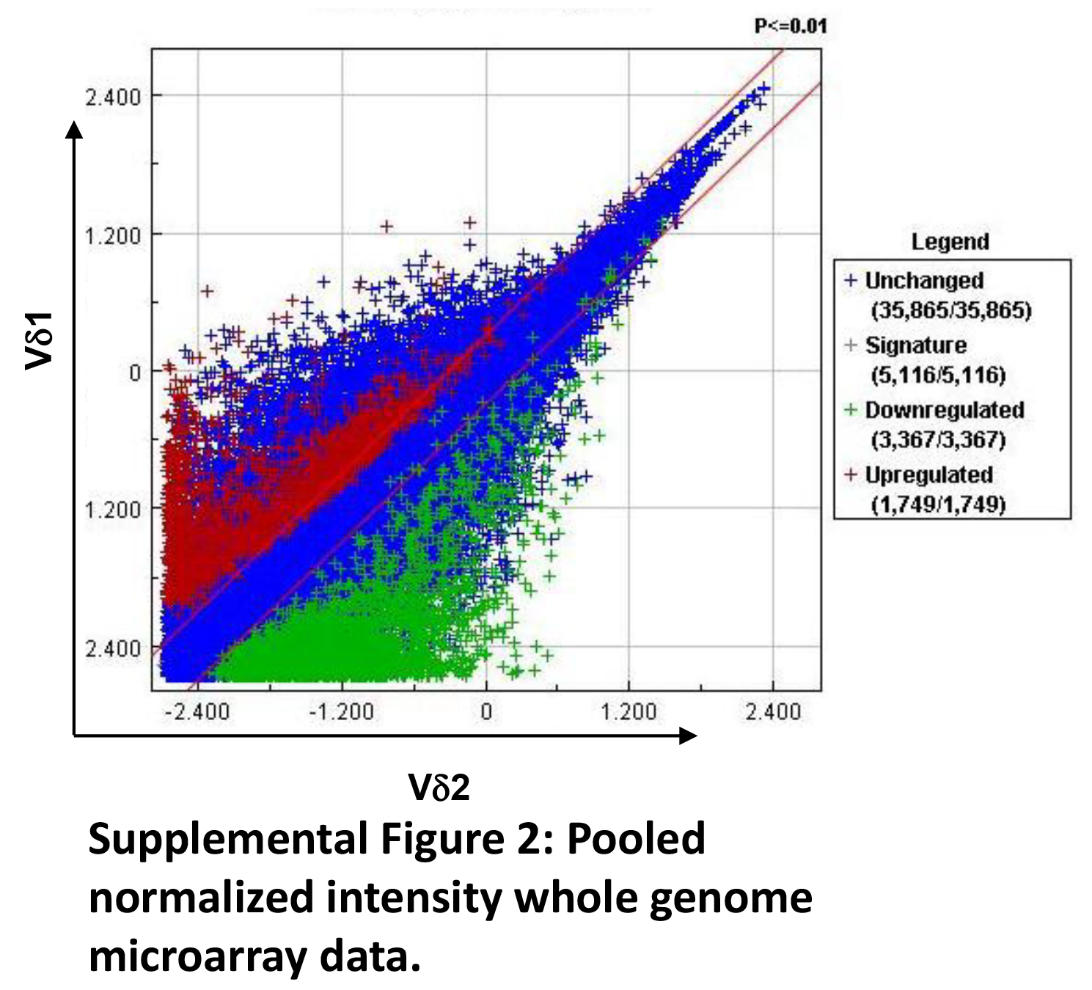


**Supplemental Figure 2.** Pooled normalized intensity whole genome microarray data.
